# Neural correlates of standing imagery and execution in Parkinsonian patients: The relevance to striatal dopamine dysfunction

**DOI:** 10.1371/journal.pone.0240998

**Published:** 2020-10-28

**Authors:** Yutaro Mori, Etsuji Yoshikawa, Masami Futatsubashi, Yasuomi Ouchi

**Affiliations:** 1 Department of Biofunctional Imaging, Preeminent Medical Photonics Education & Research Center, Hamamatsu University School of Medicine, Hamamatsu, Japan; 2 Central Research Laboratory, Hamamatsu Photonics K.K., Hamamatsu, Japan; 3 Hamamatsu PET Imaging Center, Hamamatsu Medical Photonics Foundation, Hamamatsu, Japan; 4 Hamamatsu Medical Center, Hamamatsu, Japan; CNRS, FRANCE

## Abstract

It has been reported that the cerebellar vermis is equally involved in both motor imagery about axial movement and the actual execution of postural balance in healthy human subjects, but this finding is yet to be explored in Parkinson’s disease (PD). We therefore investigated the neuronal responses during observation of standing posture, imagination of standing and the assumption of an upright posture in ten drug-naïve PD patients using positron emission tomography (PET) with [^15^O]H_2_O and evaluated dopamine dysfunction by measuring the level of dopamine transporter binding of [^11^C]CFT. Within-group statistical parametric mapping (SPM) analysis showed similar cerebellar activation during imagination of standing and its real execution between the PD and control groups (12 healthy subjects); i.e., increases in regional cerebral blood flow (rCBF) were observed in the anterior cerebellar vermis during motor imagination and the posterior vermis during standing. A comparison between the groups showed that the motor execution of standing significantly activated the superior part of the posterior vermis (declive VI) and the paracentral sulcus region in the PD patients, while the prefrontal cortices were deactivated during standing (p<0.001 uncorrected). Correlation analysis within the PD group revealed that the postural rCBF increases in the cerebellar vermis (pyramis) were negatively correlated with putaminal [^11^C]CFT binding (p<0.01, r = 0.94) and that the postural rCBF reductions in the orbitofrontal cortex were positively correlated with caudate [^11^C]CFT binding (p<0.05, r = 0.70). These results suggest that while the neural circuits for postural imagery and execution are intact in PD, standing performance, which requires more recruitment of dopaminergic control, may result in compensatory overstimulation of the cerebellar vermis and paracentral foot area in PD patients. Hyperactivity in these areas along with mesocortical hypofunction may be pathophysiological aspects of postural control in PD patients. Hence, our findings would help understand the modifications observed within the neural networks in relationship with postural performance, and possible compensatory mechanisms in PD.

## Introduction

Parkinson’s disease (PD) is a neurodegenerative disorder characterized by dopaminergic cell attrition, and its symptoms are characteristic of impaired motor function including bradykinesia, tremor and balance and gait disturbance [1]. While a disturbance in postural balance develops at a later stage of PD [1], the development of postural instability varies among patients [2]. In addition to the motor deterioration, some PD patients suffer from cognitive dysfunction (e.g. disturbed executive function) [2]. Indeed, an impairment of executive function was reportedly associated with poorer performance of balance in PD patients [3]. Previous reports indicate that dopaminergic degeneration leading to the disruption of the basal ganglia-thalamocortical and brainstem systems may be responsible for this standing-induced exacerbation [4,5]. A single photon emission tomography (SPECT) study showed that neuronal activity was increased in the cerebellar vermis, and the insular and cingulate cortices during walking in PD patients [6], indicating that compensatory hyperactivation occurs in these postural and attentional brain regions due to abnormal dopamine flow. In addition, a locomotion-induced reduction in regional cerebral blood flow (rCBF) was observed in the frontal motor areas, which reappeared during upper extremity movement [7]. Thus, axial movement-related functional hyper- and hypo-alterations may be characteristic of the degeneration of the brain in PD. The question remains as to whether cognitive function is associated with the load (stress) of postural standing in early-stage PD patients with no marked postural instability.

Our previous positron emission tomography (PET) study showed significant postural activation in the cerebellar vermis during standing and hyperactivation in the prefrontal cortex during eye-closing or motor imaging of standing in healthy human subjects [8]. Previous human lesion studies reported that medial cerebellar lesions disturbed balance and gait, while lateral cerebellar lesions impaired motor coordination of the distal extremities [9,10]. These findings indicate that the cerebellar vermis plays a major role in control of axial movement in humans; i.e., the cerebellar vermis is not only important for postural motor control but also non-motor perception because it is also involved in kinesthetic imagination (motor imagery) by serving as the simulator of an action without any accompanying body movement during execution-related imagery [11]. Indeed, imagined standing and locomotion preferentially activate the medial cerebellar region in healthy subjects [12]. Thus, these findings indicate that the cerebellar vermis in the healthy brain is involved in higher brain function irrespective of mental activity or actual motor performance. However, it is yet to be explored whether this rule can be applied to the degenerated PD brain.

As shown in behavioral improvement using external sensory cues in PD [4], motor-related brain function is compensated for by additional processing or overactivation of the extrastriatal brain regions [13,14]. For example, sequential finger movements in the affected hand activate the lateral premotor cortex, cerebellum, and primary sensorimotor cortex in PD. Likewise, it can be extrapolated that standing behavior under balance impairment might cause overactivation of the motor control system in PD patients. Methodological limitations have so far prevented the examination of compensatory brain responses related to postural imbalance or falls; however, our refined PET camera [15,16] is capable of depicting alterations in the brains of PD patients under such circumstances.

Here, we aimed to clarify the relation of cognitive function (motor imagery) with the load of standing posture and discuss the possibility of the additional pathophysiology of PD by examining dopamine dysfunction and neuronal activations in parallel using our PET system.

## Materials and methods

### Subjects

This study was approved by the Ethics Committee of Hamamatsu Medical Center, and written informed consent was obtained from all participants prior to taking part. A total of 22 subjects: 12 healthy subjects (5 males and 7 females, 9 right-handed and 3 left-handed, mean age: 51.2 ± 9.2 y) and 10 right-handed PD patients (3 males and 7 females, mean age: 57.1 ± 6.2 y) participated in this study. Healthy subjects were recruited via in-house advertisement and screened for exclusion criteria: regular intake of medicines, a history of psychiatric or neurological diseases, and contra-indications to MRI and PET scanning (claustrophobia, metallic implants, pregnancy). Patients with PD were recruited through hospitals and clinics related to Hamamatsu Medical Center and screened for the same exclusion criteria with one important additional criterion: a history of any kind of dopamine therapy. In order to exclude the possibility of dopaminergic drug effect on dopamine PET data and CBF responses either under the on-state or off-state condition, we recruited all drug-naïve PD patients rated as the Hoehn-Yahr stage II or less, who did not suffer from significant postural instability but bradykinesic foot movoment. The patients were assessed using the Unified Parkinson’s Disease Rating Scale (UPDRS) and Mini-Mental State Examination (MMSE) (Table 1). L-Dopa treatment after PET examination was effective for parkinsonian symptoms in all PD patients. As shown in Table 1, no patients with cognitive impairment were included.

**Table 1:** Clinical characteristics of Parkinson’s disease patients.

No	age sex	DD (y)	H&Y	Affected side	MMSE	UPDRS	Medication
						men/act/motor	
1	57 M	0.8	2	R ≤ L	28	4 / 9 / 13	naïve
2	60 F	2.0	2	R ≥ L	30	2 / 3 / 11	Anti-hypertensive
3/	58 M	1.5	2	R = L	30	4 / 6 / 6	naïve
4/	59 F	1.1	2	R = L	29	4 / 6 / 10	naïve
5	72 F	2.1	2	R = L	30	2 / 5 / 9	naïve
6	57 F	1.0	1	R > L	28	4 / 5 / 13	naïve
7	61 F	3.0	2	R ≤ L	29	4 / 8 / 12	Thyroid hormone
8	55 M	2.8	2	R = L	30	2 / 6 / 7	naïve
9	59 M	1.1	2	R = L	29	3 / 7 / 11	naïve
10	56 M	0.6	1	R < L	30	1 / 3 / 10	naïve

DD: Disease duration (y), H&Y: Modified Hoehn & Yahr disability score (1–5), R: Right, L: Left, MMSE: Mini-Mental State Examination, UPDRS: Unified Parkinson’s Disease Rating Scale (me/act/motor: me: mentation, behavior and mood, act: activities of daily living, mo: motor examination).

### Behavioral task

The participants were allowed several attempts to complete the tasks given in the PET scans prior to the measurement. First in the supine condition, we used a computer screen placed 1 meter before each subject, whilst they were lying on the scanner’s couch. The subjects were instructed to stare at a marker of a human silhouette that was standing in front of the door of our PET room displayed on the computer screen (Task1: Stare, S1A Fig), and then imagine that they were standing upright while fixing their eyes on the center of the screen (Task2: Imagination). After the supine-condition task, the subjects were asked to stand upright for 2 minutes with their head fixed to the head receiver attached on the gantry surface (Task3: Stand). In this condition, the subjects stared at a target hanging lower on the back of the folded couch 1 meter in front of them (See S1B Fig).

**Fig 1:**
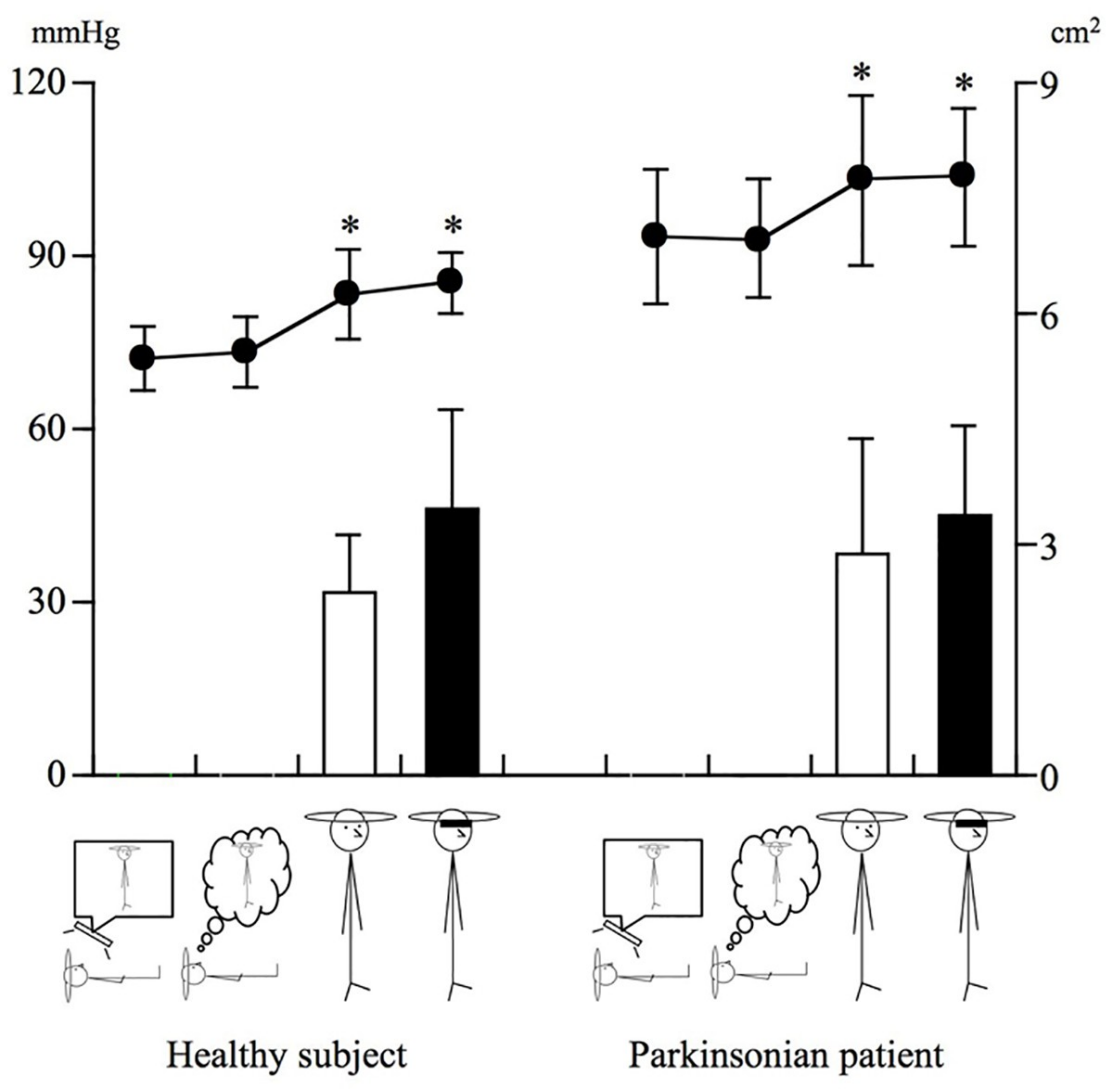
Physiological and behavioral data during tasks. After each subject practiced twice, their blood pressure and the magnitude of sway during standing with eyes open and closed were measured (See, Table 2). The data showed no significant change in the pattern of blood pressure and sway between the groups, although the sway level tended to be higher during standing with eyes open. * p<0.05, paired t-test.

In the preliminary session for evaluating physiological changes between the two groups, systemic blood pressure and behavioral stability were examined for differences in postural balance ability (as seen in Fig 1, standing with eyes open and closed). Standing with eyes closed was just for evaluation of the contribution of proprioception in balance. Regarding the magnitude of postural sway, it was evaluated by measuring the displacement of the center of foot pressure during standing upright and calculating the area of sway before and after the PET study using a stabilometer (Gravicorder GS-30, Anima KK, Tokyo, Japan).

### Imaging procedure

All subjects underwent 3-dimensional MRI just before PET measurement. Here, a static magnet (0.3 T MRP7000AD, Hitachi, Tokyo, Japan) was used with 3-dimensional mode sampling to determine the areas of the striatum in which to set the regions of interest (ROI). The MRI measures and a mobile PET gantry allowed us to reconstruct the PET images parallel to the intercommissural (ACPC) line without reslicing. Using this approach, we were able to allocate ROI on the target regions of the original PET images. (Ouchi et al., 1998).

PET study 1: Regional CBF was measured twice for each condition using a high-resolution brain PET scanner (SHR12000, Hamamatsu Photonics K.K., Hamamatsu, Japan) [15], which was capable of yielding 47 tomographic images simultaneously. Following a 10-min transmission scan for attenuation correction using a ^68^Ge/^68^Ga source with the subject’s head fixed by a radiosurgery-purpose thermoplastic facemask, a 60-s emission scan was acquired from the time when the radiotracer first entered the cerebral circulation after an intravenous bolus injection of 300 MBq of H_2_^15^O had been administered. [17,18].

PET study 2: Within a week of the completion of the PET paradigm study 1, a dopamine PET study was performed in the parkinsonian group. Serial scans (time frames: 4x30s, 20x60s, 14x300s) lasting 92 min were performed in the supine position with intermittent arterial blood sampling after a slow bolus injection of 350-MBq of dopamine transporter marker [^11^C]2-ß-carbomethoxy-3ß-(4-fluorophenyl) tropane ([^11^C]CFT). The detail was described elsewhere [19]. Briefly, to determine radioactive metabolites, additional arterial blood samples were drawn at 1, 5, 20, 30 and 45 min after [^11^C]CFT injection and analyzed using thin-layer chromatography and a storage-phospher-screen bioimaging analyzer (BAS-1500, Fuji Film, Tokyo, Japan). The free metabolite-corrected plasma activities were fitted to a sum of three exponentials by the nonlinear least-squares method with the non-weighted Gauss-Newton algorithm.

### Data analysis and statistics

Semicircular ROIs were drawn bilaterally over the caudate, putamen, and cerebellum on the MR images as described elsewhere.[20] With the application of a two-tissue compartment model using metabolite-corrected arterial and tissue time activity curves, the final measure was estimated as the binding potential (or ratio of k_3_ to k_4_) of the [^11^C]CFT in the striatum bilaterally. The value was used as a covariate in further correlation analysis. The reduction of the binding potential was additionally depicted voxelwise using statistical parametric mapping (SPM) software (Wellcome Trust Centre for Neuroimaging, London, UK, http://www.fil.ion.ucl.ac.uk/spm/). In this additional image analysis, the side of the reduction contralateral to the more affected limb was set on the right so that the symptom-induced hemispheric side difference was eliminated by flipping the images.

The CBF data were analyzed using SPM8 without flipping any images. The CBF images were first normalized to the standard stereotaxic Talairach brain atlas and smoothed with an isotropic Gaussian kernel of a full-width at half-maximum of 8 mm. The effects of global CBF were removed by normalizing each voxel count to the total brain count using proportional scaling in SPM. The between-group comparisons (PD vs. Normal) of CBF in each condition were performed by regarding the stabilometer data as confounding covariates using a t-test at each voxel. Clusters of a maximum of 50 contiguous voxels with a p value of <0.05 corrected for multiple comparisons using the FDR method were considered statistically significant.

## Results

### Physiological and behavioral variables

As seen in Fig 1 and Table 2, although blood pressure was higher during standing than lying (p<0.05, paired t-test), there was no significant difference between the groups. Due to this, we excluded the effects of physiological and behavioral responses on the outcome of brain activation during tasks when making further comparisons between groups.

**Table 2:** Physiological and behavioral data for each condition in two groups.

Parameter	State	Condition	PD	NC
Mean blood pressure	Supine	Viewing	89.9±11.0	71.3±4.3
(mmHg)		Imagination	91.9±13.8	71.8±5.4
	Standing	Eye open	103.0±14.3	83.2±7.8
		Eye closed	103.5±12.0	85.1±5.3
Stabilometer	Standing	Eye open	2.9±1.9	2.4±1.5
(cm^2^)		Eye closed	3.1±1.4	3.5±1.6

Values are expressed as mean ± SD. PD: Parkinson’s disease, NC: Normal control.

### Task-induced brain activation in each group

**Fig 2:**
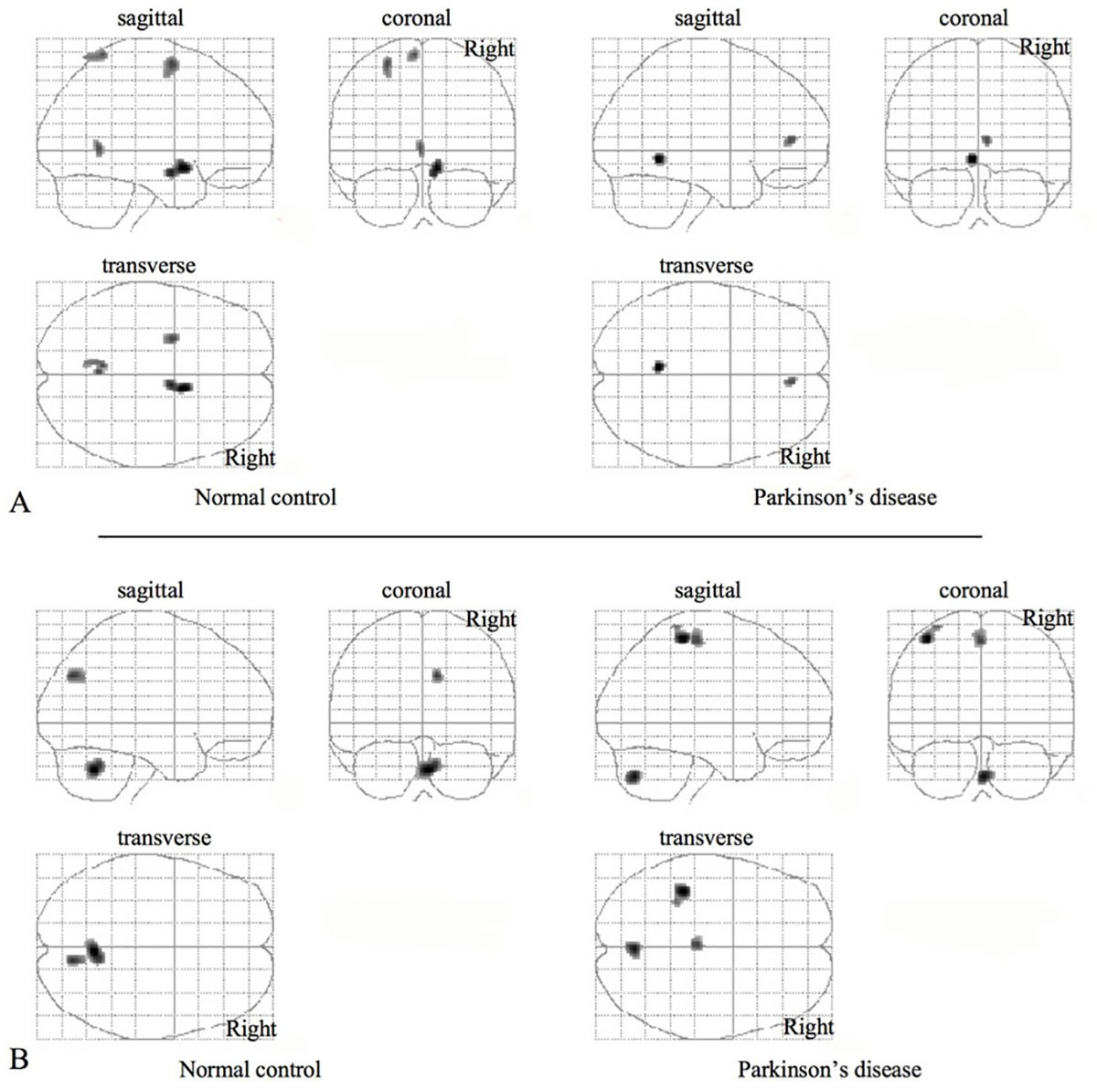
Statistical parametric maps (glass brains) in within-group comparisons. Increases in rCBF were observed during imagination of standing vs. viewing (A), during standing vs. imagination of standing (B). The detail of coordinates is described in the Table 3.

A comparison of imagining standing with staring in the supine condition (Task2 –Task1) showed a significant rCBF increase in the cerebellar vermis (culmen) in both the PD and NC groups (Fig 2 and Table 3) and different parts of the frontal and parietal regions. A comparison of standing with imagining standing (Task3 –Task2) showed a significant rCBF increase in the left somatosensory hand area (Brodmann area or BA 2), cerebellar vermis (pyramis), and left paracentral foot region (BA 6) in the PD group and the cerebellar vermis (pyramis) and right cuneus (BA 19) in the control group. The commonly activated region between the two groups was the cerebellar vermis during motor imagery and the execution of an upright posture. All the right-handed PD patients tended to grip the supporter pipe with their right hand to maintain balance during standing, which might have been the reason for the focal activation seen in these patients in the sensory area on the left-side of the brain. Furthermore, there is a possibility that allowing to grip the supporter pipe might affect feelings of postural instability and might have significantly modified their postural control performance.

**Table 3:** Brain areas with significant rCBF increase in within-group comparisons in each group.

Group	Contrast	Brain region	Coordinates (x y z)	*Z*
PD	Imagination—Stare	L cerebellar vermis (Culmen IV)	-4–50–4	4.51
		R ACC (BA: 32)	6 44 6	3.92
	Stand—Imagination	L postcentral gyrus (BA: 2)	-40–36 62	5.15
		R cerebellar vermis (Pyrumis VIII)	2–72–40	4.96
		L paracentral lobule (BA: 6)	-2–26 60	4.76
NC	Imagination—Stare	R subcallosum (BA: 25)	10 8–14	4.40
		L middle frontal gyrus (BA: 6)	-26–2 62	3.95
		L cerebellar vermis (Culmen V)	-2–56 0	3.89
		L superior parietal lobule (BA: 7)	-6–52 68	3.87
	Stand—Imagination	R cerebellar vermis (Pyrumis VIII)	4–58–34	5.66
		R cuneus (BA: 19)	10–74 34	5.10

BA: Brodmann area, R: Right, L: Left.

### Between-group comparison of rCBF responses and their relevance to clinical variables in the PD group

**Fig 3:**
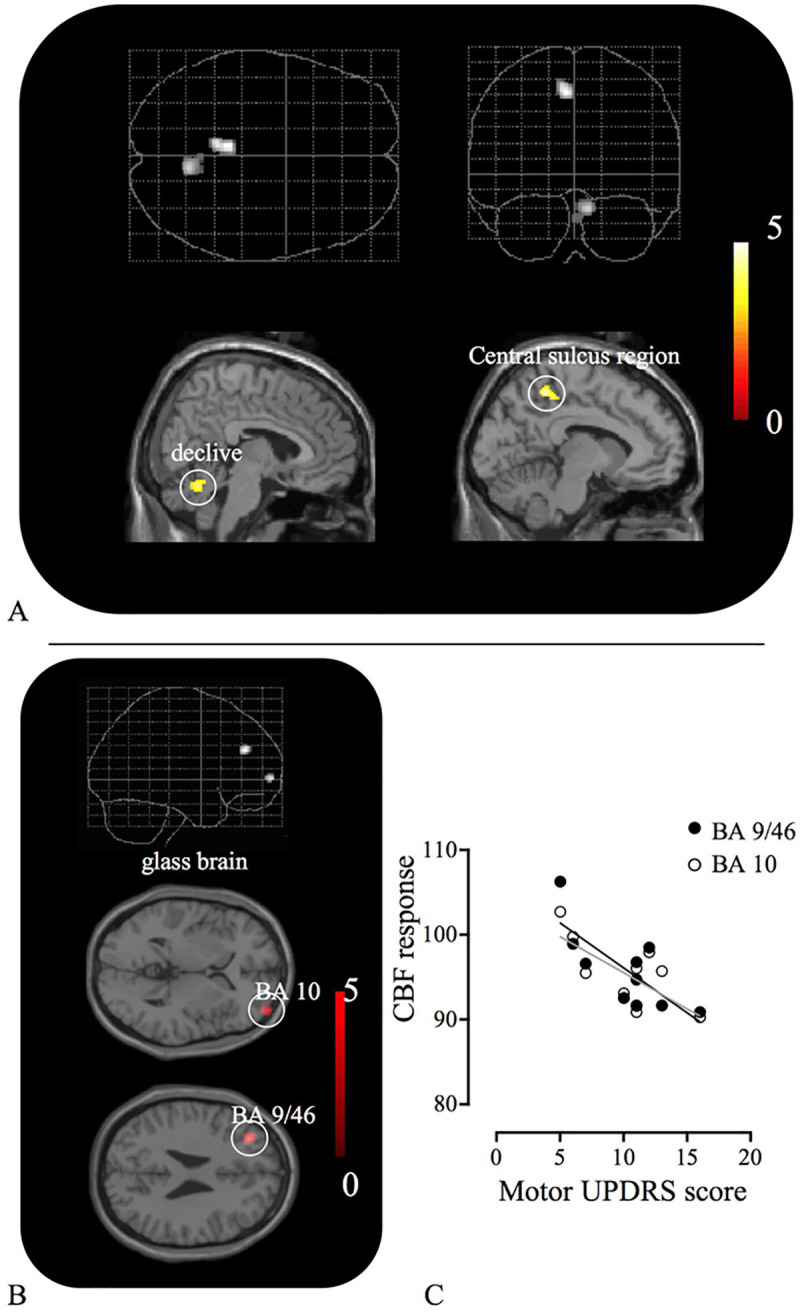
Statistical parametric maps in between-group comparisons and clinico-functional correlation. Significant increase in rCBF was found in the central sulcus region and cerebellar vermis (A), and significant rCBF reductions in the bilateral prefrontal cortices (B) during standing in PD group compared with normal group. The CBF responses in these prefrontal regions were negatively associated with motor UPDRS score (C). The detail of coordinates is described in the Table 4.

As shown in Table 4, subtraction of the standing-induced responses seen in the NC group from those observed in the PD group revealed significant increases in the cerebellar vermis (declive) and paracentral sulcus region (foot area) in the PD group (Fig 3A), and the opposite subtraction revealed a significant rCBF reduction in the bilateral prefrontal cortices (Fig 3B) in the PD group. There were no significant changes regarding motor imagery of standing between the two groups in this study. In the PD patients, the postural rCBF reduction in the prefrontal regions (right BA10 and left BA9/46) was negatively correlated with the motor UPDRS scores (Fig 3C). This indicates that postural deactivation in the prefrontal cortex is related to the deterioration of motor performance in PD patients.

**Table 4:** Brain regions with significant rCBF changes during standing in PD compared with NC.

Response	Brain region	Coordinates (x y z)	*Z*
Increase	L paracentral gyrus (BA: 5)	-6–36 52	4.19
	R cerebellar vermis (Declive VI)	8–62–22	3.91
Decrease	L middle frontal gyrus (BA: 9/46)	-36 40 26	4.62
	R middle frontal gyrus (BA: 10)	38 62 2	4.40

BA: Brodmann area, R: Right, L: Left.

### Correlation of striatal [^11^C]CFT binding with postural rCBF responses in the PD group

**Fig 4:**
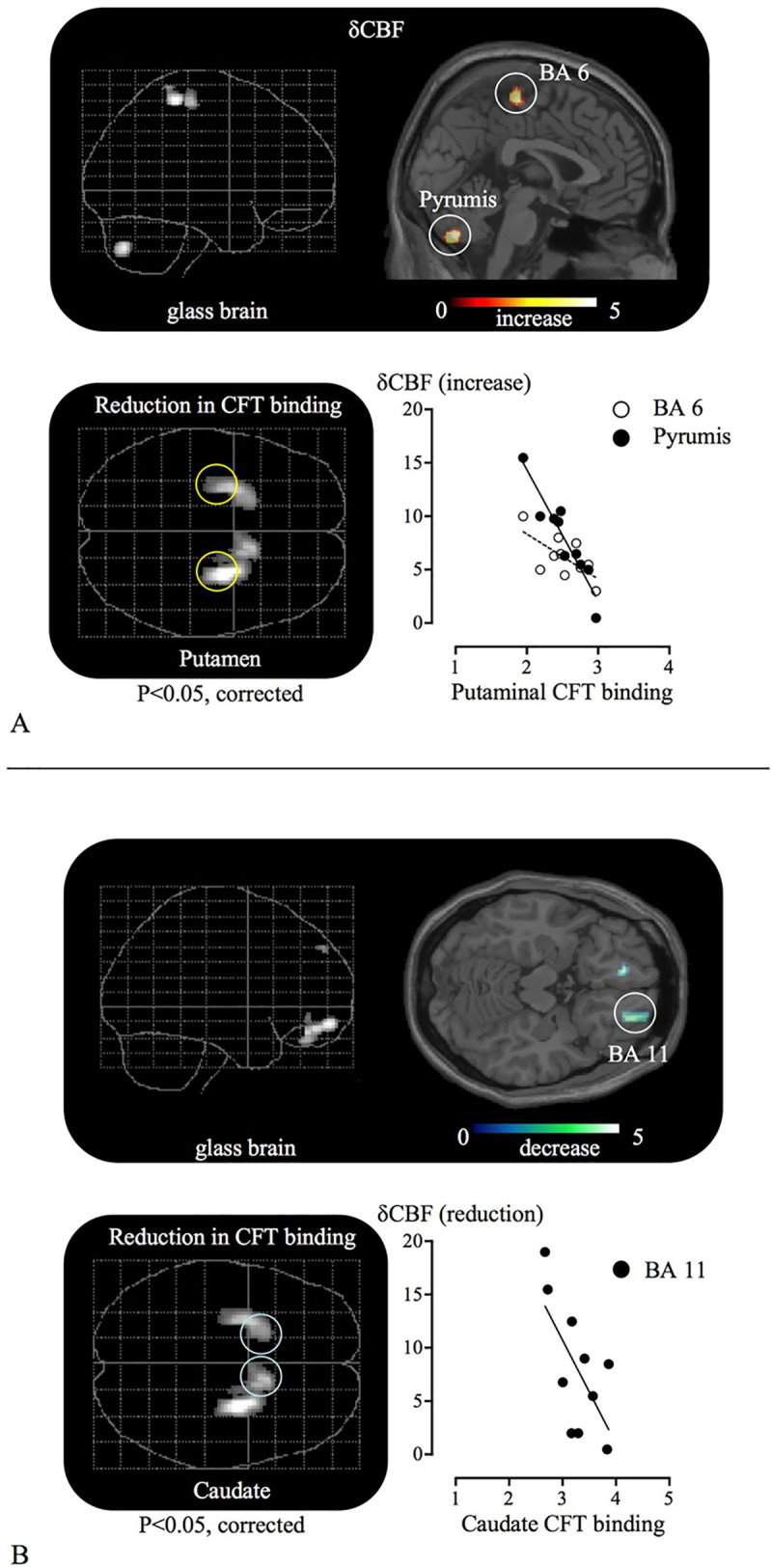
Correlation of dopamine dysfunction and postural brain responses in PD. A: Brain responses in the cerebellar vermis with postural hyperactivation in the within-group comparison significantly correlated negatively with CFT binding in the putamen (Scattergram, black circle), where significant reduction in striatal CFT binding was observed (also see Table 5). B: Brain responses in the orbitofrontal cortex with postural hypoactivation in the within-group comparison significantly correlated positively with CFT binding in the caudate (Scattergram, black circle).

As shown in Table 5, the levels of [^11^C]CFT binding potential in the PD group were significantly lower than those in the normal control group as found in our previous study [21]. This finding was illustrated in a SPM glass brain with a statistical threshold of 0.05 corrected for multiple comparisons (Fig 4A and 4B). Correlation analysis showed that the postural rCBF increase in the cerebellar vermis (pyramis) was negatively associated with the putaminal [^11^C]CFT binding (Fig 4A closed circles; p<0.01, y = -12.3x + 38.9, r = 0.94), while the postural rCBF reduction in the orbitofrontal cortex (BA 11) was associated with a decrease in caudate [^11^C]CFT binding (Fig 4B closed circles; p<0.05, y = —9.7x + 39.9, r = 0.70).

**Table 5:** Binding potentials for [^11^C]CFT in the striatum.

	Caudate		Putamen	
	Right	Left	Right	Left
PD	3.06±0.33*	3.15±0.32*	2.43±0.43**	2.42±0.27**
NC†	3.92±1.13		4.14±0.95	

Values are expressed as mean (SD). The averaged values of normal control (†) are those obtained in our previous study [21].

**p<0.01

*p<0.05 vs. normal control. NC: Normal control, PD: Parkinson’s disease.

## Discussion

We for the first time showed postural overactivation of the cerebellar vermis and primary motor area during standing in PD patients, the degree of which was negatively associated with dopaminergic impairment in the motor striatum and putamen. Interestingly, this vermal activation was stronger than the activation shown in a series of gait studies using SPECT [6,22] and a postural balance study using PET [8]. The negative correlation between vermal activation and striatal dopamine deficiency in the present study suggests a compensatory mechanism in which additional motor areas that subserve axial movement are recruited to compensate for the striatal deficiency in PD brains. This is in line with the brain overactivation observed in the motor and perceptual brain regions of PD patients during finger movement on the bradykinetic side, which compensates for the basal ganglia deficiency caused by PD [23].

The hyperactivation of the cerebellum in PD has been reported in various motor activation studies using SPECT [24] and fMRI [25,26], in which robust activation was found during finger or hand movement in the lower anterior to upper posterior cerebellar region including the anterior-posterior vermis (lobule V~VI). Likewise, the present result showed significant activation during standing in lobule VI in a comparison of PD patients with normal controls (See Fig 3A, Table 4) although the tasks were different. The role of lobule VI has yet to be elucidated, but a meta-analysis of neuroimaging studies on cerebellar functional topography showed that sensorimotor tasks often activate the anterior lobe (lobule V), lobule VI, and additional loci in the posterior lobe (lobule VIII) [27]. A recent patient study showed that a cerebellar lesion extending into lobule VI caused a greater impairment of gait in patients with cerebellar stroke, but the lesion itself generated minimal motor impairment [28]. Since it is generally accepted that the cerebellum has dual functional systems; i.e., the anterior lobe for sensorimotor processing and the posterior lobe for cognitive/emotional processing [29], lobule VI may function as a mediator that integrates multimodal input from the cerebral cortex and spine, just like the premotor cortex in the frontal lobe as suggested by Schmahmann et al. [28]. Indeed, overactivity of the premotor cortex was observed during paradoxical gait in PD patients [30]. The suggestion of an integral role for lobule VI is supported by another study that showed that the posterior vermis including lobules VI and VII is involved in the generation of slow eye pursuit movement in humans [31]. Therefore, the question is why did lobule VI show hyperactivation during standing in PD patients in the present study. One interesting finding of Rascol et al. was that patients with PD receiving dopaminergic treatment were free from cerebellar overactivation [24], suggesting that the central dopaminergic deficit is responsible for this abnormal response. Our findings are compatible with this because cerebellar activation in the posterior vermis was found to be inversely correlated with dopamine dysfunction (the reduction in dopamine transporter binding in the putamen) (Fig 4A). Thus, it is likely that standing-evoked posterior vermal activation reflects overloading of its multimodal processing capacity to compensate for the loss of basal ganglia control in PD.

In the present study, the sensorimotor foot area in the cerebral cortex was also activated during standing in PD patients in this study. It was reported that PD patients with more severe rigidity showed a greater fMRI signal increase in the contralateral primary motor cortex during finger movement and that there was a negative correlation between the fMRI responses between the contralateral putamen and ipsilateral cerebellum [26]. This overshoot of motor activity during motor tasks may result from the loss of control of the nigrostriatal-thalamo-cortical system. However, it is unclear whether this motor-induced S1/M1 overactivation is caused directly by dopamine deficiency because in our sensorimotor standing task, the CBF response was not significantly correlated with the reduction in dopamine transporter binding (Fig 4A). A functional correlation MRI study showed functional connectivity between the motor cortex and anterior cerebellum and lobule VIII [32]. Thus, the greater sensorimotor activation seen during standing in PD patients in the current study might reflect a compensatory response to dopamine deficiency. Although we did not measure CBF response during standing with eyes closed in this study, it is likely that closing the eyes would require cerebellum involvement to maintain stability. Then, a further study is needed to clarify the brain activation evoked during standing with eyes closed because in the upright position the withdrawal of visual input requires proprioceptive and cerebellar information to compensate and maintain stability.

The prefrontal cortex is connected anatomically and functionally to the striatum. In the present study, the magnitude of the prefrontal activation (CBF response) was found to be positively correlated with the motor severity of parkinsonism (Fig 3B and 3C). It is now widely accepted that the dorsolateral prefrontal cortex is involved in the selection or programming of novel movements in healthy subjects [33,34]. In PD patients, however, medial and dorsolateral prefrontal activity was found to be lower during a self-paced joystick movement than in healthy subjects [35,36]. Taken together, it is suggested that the capacity for predicting or selecting the appropriate step for a forthcoming movement during standing or walking is poor in PD patients. This is also in line with the present finding that the standing-induced reduction in neuronal responses in the orbitofrontal cortex of PD was positively associated with low dopamine transporter binding in the caudate (a functional part of the mesocortical dopamine projection system in PD patients [19]) (Fig 4B). In other words, as suggested in a recent imaging study showing reduced activity in the dorsolateral and ventrolateral prefrontal cortices during tasks requiring the caudate in PD patients [37], the compromised mesocortical dopamine system in PD patients may generate a cognitive defect during standing or axial movement. Although the behavioral capacity measured by posturography was the same between the two groups in the present study, a latent cause of this pathophysiological change may already exist in PD brains.

There are a couple of limitations. First, the head holder attached to the scanner’s gantry was used to immobilize the subject’s head during standing in the present study, which indicated that standing here was not the same as free standing without support. Currently, though, no immobilization technique during standing exists. Second, no inclusion of patients with postural instability (H&Y 3 or more) cannot provide data for primary involvement of postural disturbance in PD. Third, no measurement during standing with eyes closed did not provide brain activation data under the influence of postural sway for proprioceptive control in balance in PD.

## Conclusions

In conclusion, we showed abnormal neuronal responses during standing in mildly bradykinetic PD patients in the present study. These early PD patients used their neuronal circuits during motor imaging of standing in a similar manner to healthy subjects. However, standing generated increased activation in the superior part of the posterior cerebellum and the paracentral foot area in the PD patients. This suggested compensatory overactivation in these posture-related areas against the dopaminergic deficiency caused by PD. The hyperactivation in these areas and reduced mesocortical dopamine function reflects a dynamic aspect of posture-related pathophysiology in PD.

## Supporting information

S1 Fig A scene of PET examination during standing. A subject looks at a figure displayed on the monitor (A) and stare at a marker placed 1 meter before the standing point (B). A maximum of 2 minutes was required to stand still.
(JPG)
