# Supplementary figures and images for: Neural correlates of standing imagery and execution in Parkinsonian patients: The relevance to striatal dopamine dysfunction
Source: PLoS One. 2020 Oct 28;15(10):e0240998. doi: 10.1371/journal.pone.0240998 (PMC7592757; doi:10.1371/journal.pone.0240998)

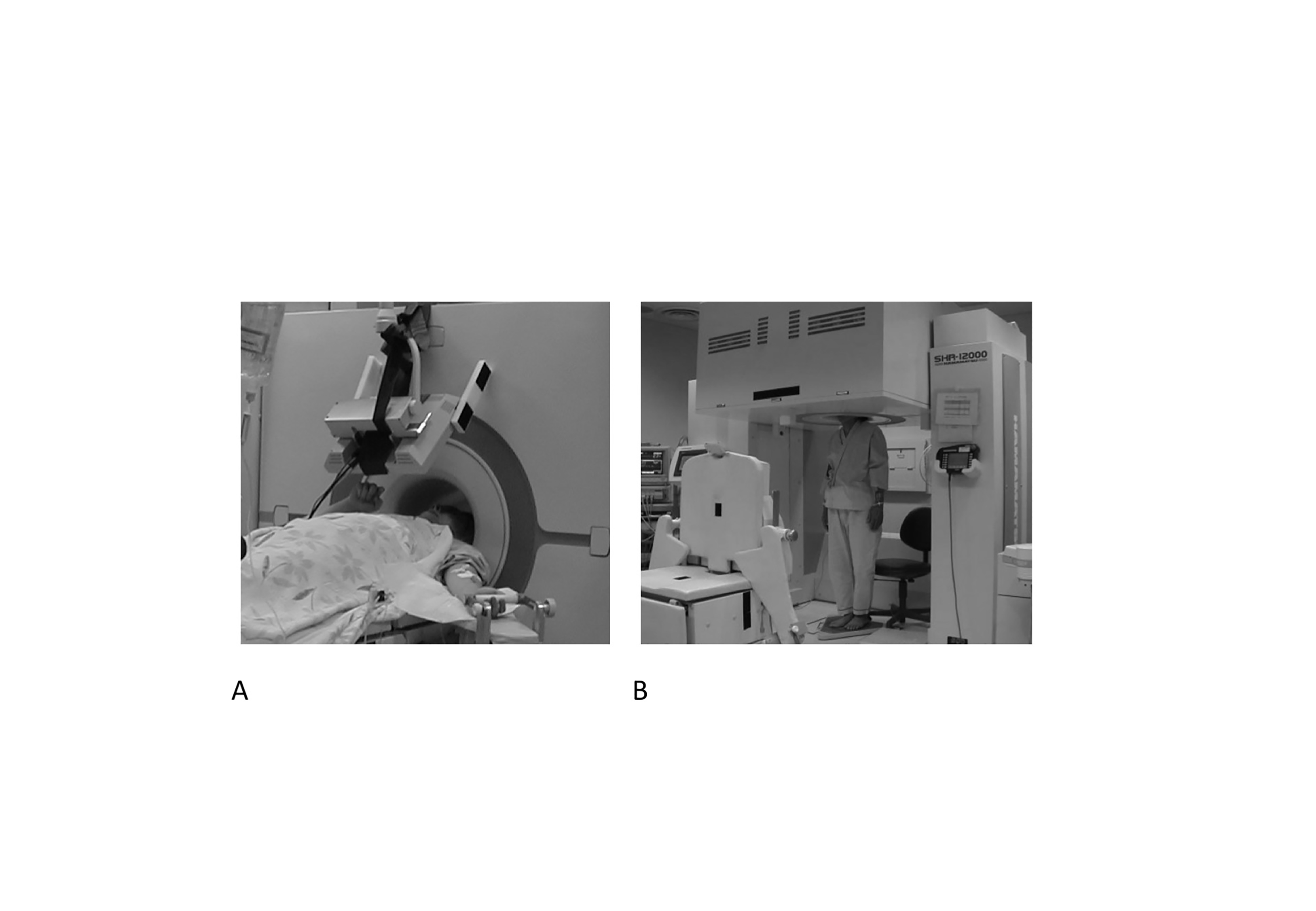

Supplement: S1 Fig — A subject looks at a figure displayed on the monitor (A) and stare at a marker placed 1 meter before the standing point (B). A maximum of 2 minutes was required to stand still. (JPG) [file pone.0240998.s001.jpg]
